# Anterior Interosseous Nerve to Ulnar Nerve Transfer: A Systematic Review

**DOI:** 10.1016/j.jpra.2022.02.007

**Published:** 2022-03-11

**Authors:** M. Thakkar, A. Rose, W. King, K. Engelman, B. Bednarz

**Affiliations:** 1 Department of Plastic and Reconstructive Surgery, Glasgow Royal Infirmary, 84 Castle Street, Glasgow, G4 0SF; 2 Department of Genetics and Genomic Sciences, Icahn School of Medicine at Mount Sinai, New York; 3 Department of Plastic and Reconstructive Surgery, Southmead Hospital, Southmead Road, BS10 5NB

**Keywords:** Nerve transfer, Upper-limb nerve transfer, Anterior interosseous nerve, AIN, AIN to ulnar nerve transfer, Ulnar nerve, End-to-side nerve transfer, Supercharged end-to-side nerve transfer, End-to-end nerve transfer

## Abstract

**Background:**

Ulnar nerve injuries, especially high (proximal forearm) injuries, result in poor functional recovery. Peripheral nerve transfers have recently become a popular technique to augment nerve repairs and reduce the reinnervation distance before distal motor endplates irreversibly degenerate, leading to incomplete recovery.

**Objectives:**

To systematically review and analyse the recent literature regarding anterior interosseous nerve (AIN) to ulnar nerve transfers, including demographics, indications, outcomes, and complications.

**Methods:**

A search was performed using PubMed, MEDLINE, EMBASE, CINAHL, Scopus, and Cochrane databases using the keywords ulnar nerve, ulnar nerve injury, ulnar motor nerve, anterior interosseous nerve, anterior interosseous, AIN, nerve transfer, and end-to-side using a 3-component search along with the Boolean operators ‘AND’ and ‘OR’.

**Results:**

A total of 341 studies were retrieved using the search criteria. Sixteen studies met the inclusion criteria including 12 retrospective case series, 3 retrospective cohort studies, and a single randomised control trial. Nine studies involved supercharged end-to-side transfer (SETS), 6 involved end-to-end transfer (ETE), and only 1 study compared results between SETS and ETE transfers. A total of 269 patients underwent nerve transfers. In the ETE subgroup, the average time to nerve transfer was 7 months, with a mean follow-up period of 24.5 months. Post-procedure, 100% (37/37) patients recovered intrinsic function of BMRC ≥1, and the average recovery time was 3.6 months. A total of 85% of patients recovered intrinsic function of BMRC ≥3. In the SETS group, the average time to nerve transfer was 2.5 months. The average follow-up in this cohort was 13.2 months. About 93% (145/156) recovered the intrinsic function of BMRC ≥1, and the average time to recovery was 7 months. About 75% of patients recovered the intrinsic function of BMRC ≥3 in their first dorsal interossei.

**Conclusion:**

AIN to ulnar nerve transfer carries low morbidity, and there is low quality evidence to suggest recovery of intrinsic muscle function compared with conventional primary repair techniques. The supercharged end-to-side transfer (SETS) seems to be more favourable compared with end-to-side transfer. Outcome measurements are highly variable amongst studies, making standardisation difficult. Results of further trials are highly anticipated in this exciting field of peripheral nerve surgery.

## Introduction

Ulnar nerve injuries have devastating consequences, which often result in poor functional recovery especially in adults^1^. The ulnar nerve is critical to hand function because it is responsible for the majority of hand motor function via the intrinsic muscles and provides sensation to the ulnar side of the hand. The disruption of the ulnar nerve leads to imbalance between flexors and extensors causing loss of lateral pinch strength and digital dexterity and results in a claw hand deformity. The degeneration of the distal motor endplates of the intrinsic muscles of the hand irreversibly before reinnervation from the regenerating ulnar nerve axons from the repair site because of the considerable gap and distance leads to incomplete functional recovery. This provides a treatment dilemma to promptly reinnervate distal targets to preserve motor endplate function and potentially improve functional outcome after these injuries.

Primary repairs, even with nerve grafts, can result in sensory recovery; however, the recovery of motor intrinsic function is poor^2^^,^^3^, especially in more proximal (high) ulnar nerve injuries. To overcome this considerable regeneration distance, distal nerve transfers were proposed from the anterior interosseous nerve (AIN) to the motor branch of the ulnar nerve in an end-to-end fashion^4^. However, this resulted in end targets being innervated solely by the donor nerve (AIN) and not the regenerating ulnar nerve. The supercharged end-to-side (SETS) transfer involves the donor nerve (AIN) being coapted end-to-side through a perineural window onto the motor branch of the ulnar nerve to establish distal endplate reinnervation within a shorter time frame while the more proximal repair regenerates, leading to ‘double innervation’. This was first clinically described by Barbour et al.^5^

The aim of this study was to review the existing literature and analyse the demographics, indications, outcomes, and complications of either end-to-end or supercharged end-to-side AIN to ulnar nerve transfers for ulnar nerve injuries and compression.

## Methods

A systematic review was conducted according to the Preferred Reporting Items for Systematic Reviews and Meta-Analyses (PRISMA).^6^

### Eligibility

The inclusion criteria for this review included studies that featured (1) end-to-end (ETE) or super charged end-to-side transfer (SETS) of the anterior interosseous nerve to the motor branch of the ulnar nerve in cases of ulnar nerve injuries or compressions; (2) studies that included indications, outcomes, and complications of the intervention; (3) lesions of the ulnar nerve at the level of the proximal forearm or more proximal; and (4) articles in English language. Single participant case reports were excluded along with reviews, on-going studies, animal studies, and cadaveric studies.

### Outcomes

The primary outcome measures were (1) number of patients with return of hand intrinsic muscle function of British Medical Research Council (BMRC) ≥1; (2) percentage of patients with return of hand intrinsic function of BMRC ≥3; and (3) any complications including donor site morbidity (pronation weakness).

Secondary outcome measures were (1) time to nerve transfer; (2) time to recovery; (3) post-operative grip strength; (4) post-operative key pinch strength; (5) claw correction; and (6) follow-up duration.

Because of the two different methods of anterior interosseous nerve transfer (ETE vs SETS), outcomes were divided into two subgroups. A further subgroup was included since a single study directly compared outcomes of end-to-side versus SETS AIN transfer in patients with compressive ulnar nerve neuropathies at the elbow.^7^

No result (NR) was used when data were not available or could not be extrapolated. Intrinsic muscle function was determined by physical examination in majority of cases according to the BMRC scale. Methods to measure time-to-recovery were highly variable and included physical examination or electrophysiological studies. Grip strength was measured post-operatively in comparison with the ipsilateral hand pre-operatively or post-operative comparison to the contralateral hand. There was a distinct lack of standardised reporting outcomes amongst studies.

### Search Strategy and Selection of Studies

An electronic search was performed on all published articles related to AIN to ulnar nerve transfers on PubMed, MEDLINE, EMBASE, CINAHL, Scopus, and Cochrane databases. Healthcare Databases Advanced Search (HDAS https://hdas.nice.org.uk/) was used to search PubMed, MEDLINE, Embase, and CINAHL databases. Scopus and Cochrane databases were searched independently. All databases were searched from inception to July 2021. The search terms used were ‘ulnar nerve’, ‘ulnar nerve injury’, ‘ulnar motor nerve’, ‘anterior interosseous nerve’, ‘anterior interosseous’, ‘AIN’, ‘nerve transfer’, and ‘end to side’. Terms were combined using the Boolean operators ‘AND’ and ‘OR’ in a 3-component search.

The abstracts retrieved from the search were imported on to Covidence (https://www.covidence.org/), an online systematic review management platform. Two reviewers independently screened the abstracts for relevance and subsequently reviewed the full texts for eligibility based on the inclusion criteria detailed above.

### Risk of Bias and Quality Assessment

There were no clear sources of bias identified. Quality assessment was performed using several tools including the Newcastle-Ottawa Scale^8^ for cohort studies and the NIH quality assessment tools^9^ for case series and controlled intervention studies (Appendix 1).

### Data Extraction and Analysis

Data were extracted after full-text review. Where no outcome measures were reported, no result (NR) was recorded. Data collected included intervention type, patient demographics, pre-operative examination, post-operative evaluation, complications, and primary/secondary outcome measures. Extracted data were pooled together as raw totals, means, or weighted averages. Levels of evidence were assigned to each study according to the Oxford Centre for Evidence-Based Medicine (OCEBM) Levels of Evidence.^10^

### Statistical analysis

No formal statistical analysis was performed on the selected studies.

## Results

**Figure 1:**
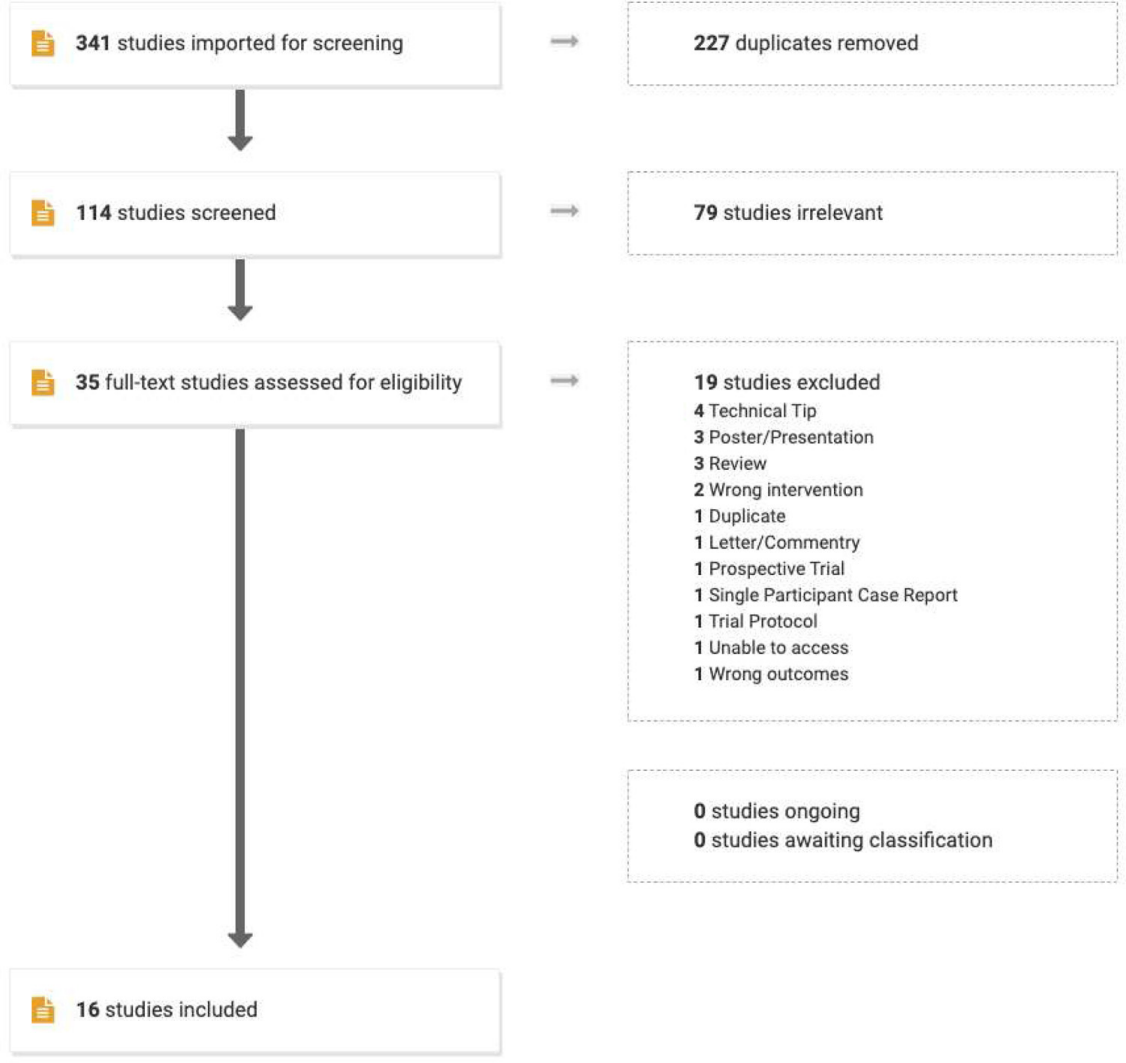
PRISMA flow diagram showing the study selection process

The search performed in July 2021 yielded a total of 341 studies. Thirty-five full-text studies were assessed for eligibility, and 16 studies met the inclusion criteria (Figure 1). Of the 16 eligible studies (Table 1), there were 12 retrospective case series, 3 retrospective cohort studies, and a single randomised control trial. Studies ranged from between 1999 to 2021. There was a single ongoing prospective trial amongst the excluded studies^11^.

**Table 1:** Showing selected studies, year of publication, study design, and assigned OCEBM level of evidence.

Author	Year	Country	Intervention	Study Design	Level of Evidence^10^
Arami^12^	2020	Israel/Brazil	End-to-end	Retrospective case series	IV
Baltzer ^13^	2016	Canada	SETS	Retrospective cohort	III
Battiston^14^	1999	Italy	End-to-end	Retrospective case series	IV
Chen ^15^	2021	Taiwan	SETS	Retrospective cohort	III
Davidge^16^	2015	USA	SETS	Retrospective case series	IV
Dengler^17^	2020	USA	SETS	Retrospective case series	IV
Doherty^18^	2020	Canada	SETS	Retrospective case series	IV
Flores^19^	2011	Brazil	End-to-end	Retrospective case series	IV
Flores^20^	2015	Brazil	End-to-end	Retrospective cohort	III
Haase^21^	2002	USA	End-to-end	Retrospective case series	IV
Head^22^	2020	Canada	SETS	Retrospective case series	IV
Jarvie^23^	2018	Canada	SETS	Retrospective case series	IV
Koriem^24^	2020	Egypt	SETS	Randomised trial	II
McLeod^7^	2020	USA	SETS or End-to-end	Retrospective case series	IV
Novak^4^	2002	USA	End-to-end	Retrospective case series	IV
Nyman^25^	2021	Sweden	SETS	Retrospective case series	IV

**Table 2:** Showing patient demographics, type of injury, level of injury, and pre-operative symptoms in end-to-end transfer studies. Lesion in continuity (LIC), no result (NR), and pre-operative (p).

Study	No of Transfers	Age	M:F	Type of Injury	Location of Injury	Symptom Duration	pWeakness	pNumbness	pPain	pAtrophy	pPositive Froment's Sign
Arami^12^	11	33.3	10:1	11 transections	5 above elbow, 2 proximal forearm, 3 infraclavicular, and 1 axilla	5 months	NR	NR	NR	NR	NR
Battiston^14^	7	32.1	5:2	4 transection, 1 iatrogenic, 2 scarring, and after primary repair	1 above elbow and 6 elbow	4 months	7	7	NR	NR	NR
Flores^19^	5	25.2	4:1	2 transection, 3 LIC	1 axilla, 1 infraclavicular, 1 elbow, and 2 proximal arm	7.4 months	5	5	NR	NR	NR
Flores^20^	15	28.2	10:5	5 transection and 10 LIC	1 axilla, 3 infraclavicular, 8 arm, and 3 elbow	7.1 months	NR	NR	NR	NR	NR
Haase^21^	2	46.5	2:0	2 transection	1 above elbow and 1 elbow	3.5 weeks	1	NR	NR	NR	NR
Novak^4^	8	38	5:3	NR	8 proximal elbow	3 months	NR	NR	NR	NR	NR
**Total or weighted mean**	**48**	**41.5**	**71% M (34/48)**	**0 Compression, 13 LIC, and 24 Transection**	**25 proximal elbow, 11 elbow, and 2 proximal forearm**	**6.8 months**	**93%** **(13/14)**	**100% (12/12)**	**-**	**-**	**-**

Nine studies used supercharged end-to-side transfer as the intervention, 6 used end-to-end transfer, and 1 study compared results between SETS and ETE AIN transfers. A total of 269 patients underwent nerve transfers amongst the 16 studies.

### End-to-end Transfer

A total of 48 patients underwent end-to-end AIN transfer. The average age was 41.5 years, and 71% of patients were male. About 60% of cases involved nerve transection as the primary injury, and the commonest location was the proximal forearm in 52% of cases. The mean symptom duration was 6.5 months, with 93% (13/14) and 100% (12/12) of patients affected by weakness and numbness of the ulnar nerve, respectively.

The average time to nerve transfer was 7 months, with an average follow-up period of 24.5 months. Post-operatively, 100% (37/37) patients recovered the intrinsic function of BMRC ≥1, and the average recovery time was 3.6 months. A total of 85% (*3 studies*) of patients recovered the intrinsic function of BMRC ≥3. The mean BMRC difference between pre-ETE transfer and post-ETE transfer was 3.1 (Table 7). Individual post-operative grip assessment is shown in Table 3. There were no post-procedural complications reported.

**Table 3:** Showing post-operative outcome measures and complications in end-to-end transfer studies. Grip (grip strength), compared with the unaffected side (cf UAS), compared with the pre-operative ipsilateral side (cf pre-op IP), key pinch strength (key), opposition (opp), and not recorded (NR).

Study	Follow-up (months)	Time to nerve transfer	Intrinsic Recovery BMRC ≥1	Time to recovery(months)	Grip % cf UAS	Grip improvement cf pre-op IP	Key % cf UAS	Key improvement cf pre-op IP	Opp % cf UAS	Claw Correction	Complications
Arami^12^	19.3	5 months	NR	NR	NR	NR	NR	NR	NR	0/11	Nil
Battiston^14^	30	4 months	7/7	2 (initial reinnervation)	70.7%	NR	71.4	NR	NR	NR	Nil
Flores^19^	20	7.4 months	5/5	NR	NR	NR	NR	NR	NR	NR	Nil
Flores^20^	24.3	7.1 months	15/15	NR	NR	NR	NR	NR	NR	NR	Nil
Haase^21^	11.5	3.5 weeks	2/2	9	NR	NR	NR	NR	NR	NR	Nil
Novak^4^	18	3 months	8/8	NR	NR	↑595%	NR	↑527%	NR	NR	Nil
**Total or weighted mean**	**24.5**	**7 months**	**100%** **(37/37)**	**3.6**	**70.7%**	**↑595%**	**71.4%**	**↑527%**	**-**	**0%** **(0/11)**	**-**

**Table 4:** Showing patient demographics, type of injury, level of injury, and pre-operative symptoms in supercharged end-to-side transfer studies. Lesion in continuity (LIC) and pre-operative (p).

Study	No of Transfers	Age	M:F	Type of Injury	Location of Injury	Symptom Duration	pWeakness	pNumbness	pPain	pAtrophy	pPositive Froment's Sign
Baltzer^13^	13	35	NR	7 transection and 6 lesions in continuity	1 upper arm, 7 elbow, and 5 proximal forearm	4.4 months	13	NR	NR	13	NR
Chen^15^	13	38.1	9:4	9 transection and 4 crush	3 upper arm, 2 elbow, and 8 proximal forearm	71.5 months	NR	NR	NR	NR	NR
Davidge^16^	55	50.5	38:17	5 transection, 23 compression, 20 LIC, 6 motor neuropathy, and 1 neuritis	4 cervical spine, 13 brachial plexus, 3 upper arm, 20 elbow, 5 proximal forearm, 4 multilevel, and 6 diffuse neuropathy	33.6 months	55	52	29	52	45
Dengler^17^	42	48	33:9	42 compression	42 elbow	31 months	42	NR	NR	42	NR
Doherty^18^	30	53	21:9	30 compression	30 elbow	NR	30	NR	NR	NR	NR
Head^22^	17	56.9	11:6	2 transection, 7 compression, 7 LIC, and neuritis 1	3 upper arm, 13 elbow, and 1 forearm	17.6 months	17	NR	NR	NR	NR
Jarvie^23^	2	57.5	1:1	2 compression	2 elbow	NR	2	2	NR	2	NR
Koriem^24^	15 (11 included)	35	13:2	15 transection	9 above elbow and 6 proximal forearm	NR	11	11	NR	11	11
Nyman^25^	2	22	2:0	2 transection	2 proximal forearm	NR	2	2	NR	NR	NR
**Total or weighted mean**	**189**	**47.5**	**75% M** **(132/176)**	**104 compression, 37 LIC, and 40 transection**	**19 proximal elbow, 116 elbow, and 27 proximal forearm**	**31.7 months**	**100%** **(172/172)**	**96%** **(67/70)**	**53%** **(29/55)**	**98%** **(120/123)**	**85%** **(56/66)**

### Supercharge End-to-Side Transfer

A total of 189 patients underwent SETS AIN transfer. The average age was 47.5 years, and 75% of patients were male. About 55% of SETS AIN transfers involved ulnar nerve compressions followed by 21% for ulnar nerve transections. A total of 61% of transfers involved pathology at the elbow. Mean symptom duration was 31.7 months. Pre-operative symptoms in reporting studies included weakness (100%), numbness (96%), pain (53%), and intrinsic atrophy (98%), and 85% had a positive Froment's sign.

The average time to nerve transfer was 2.5 months (*4 studies*). Average follow-up in this cohort was 13.2 months. A total of 93% (145/156) recovered intrinsic function of BMRC ≥1, and the average time to recovery was 7 months (*4 studies*). About 75% (7 studies) of patients recovered the intrinsic function of BMRC ≥3 in their first dorsal interosseous. Eleven patients did not recover any intrinsic function. The mean difference in BMRC between pre-SETS transfer and post-SETS transfer was 1.9 (↑159%) (Table 7). Individual post-operative grip assessments are shown in Table 6. About 4.2% of patients developed minor complications listed in Table 5.

**Table 5:** Showing post-operative outcome measures and complications in supercharged end-to-side transfer studies. Grip (grip strength), compared with the unaffected side (cf UAS), compared with the pre-operative ipsilateral side (cf pre-op IP), key pinch strength (key), opposition (opp), and not recorded (NR).

Study	Follow-up (months)	Time to nerve transfer	Intrinsic recovery BMRC ≥ 1	Time to recovery(months)	Grip % cf UAS	Grip improvement cf pre-op IP	Key % cf UAS	Key improvement cf pre-op IP	Opp % cf UAS	Claw correction	Complications
Baltzer^13^	13.5	4.4 months	11/13	2.9 months	62%	NR	52%	NR	45%	NR	Nil
Chen^15^	12	2.42 months (early)	NR	NR	82.5%	NR	83.7% (early)	NR	NR	NR	Nil
Davidge^16^	8	NR	36/39	1-12 months	NR	↑29%	NR	↑29.3%	NR	NR	Nil
Dengler^17^	11.2	NR	39/42 (FDI and ADM)	Variable	NR	↑13.95%	NR	↑28.6%	NR	NR	Allergic reaction to Dermabond, fungal rash, haematoma, and persistent elbow pain
Doherty^18^	18.6	NR	29/30	8.5 months	NR	NR	NR	NR	NR	24/30	3 minor complications
Head^22^	16.7	NR	15/17 (ADM), 16/17 (FDI)	NR	NR	NR	NR	NR	NR	NR	Nil
Jarvie^23^	18	NR	2/2	6.5 months	NR	NR	NR	NR	NR	NR	Nil
Koriem^24^	18	4 weeks	10/11	NR	NR	NR	NR	NR	NR	NR	Nil
Nyman^25^	24	1.6 months	2/2	12 months	65.5%	NR	49%	NR	NR	NR	Pronation weakness that improved
**Total or weighted mean**	**13.2**	**2.5 months**	**93%** **(145/156)**	**7 months**	**71.7%**	**↑11.8%**	**67.9%**	**↑28.9%**	**45%**	**80%**	**4.2%** **(8/189)**

**Table 6:** Showing intrinsic recovery of BMRC ≥3 amongst the ETE and SETS. First dorsal interosseous (FDI) and adductor digiti minimi (ADM).

Study	Intrinsic Recovery BMRC ≥3 (%)
**End-To-End**	
Battiston^14^	86
Flores^19^	100
Flores^20^	80
**SETS**	
Davidge^16^	70
Dengler^17^	79
Doherty^18^	73 (FDI & ADM)
Head^22^	71 (FDI) & 65 (ADM)
Jarvie^23^	100
Koriem^24^	91
Nyman^25^	100

**Table 7:** Showing the difference between pre-operative and post-operative first dorsal interosseous BMRC values and the corresponding significance values.

Study	Pre-operative BMRC	Post-operative BMRC	BMRC Difference (% difference)	p-value
**End-To-End**				
Battiston^14^	0	3.9	3.9	-
Flores^19^	0	3.6	3.6	-
Mcleod^7^	0	2.6	2.6	-
**Mean**	**0**	**3.1**	**3.1**	**-**
**SETS**				
Davidge^16^	1.3	3.0	1.7 (↑130%)	<0.0001
Doherty^18^	1.0	3.3	2.3 (↑230%)	<0.00001
Head^22^	1.1	3.2	2.1 (↑190%)	<0.002
Mcleod^7^	2.0	3.2	1.2 (↑60%)	-
**Mean**	**1.3**	**3.2**	**1.9 (↑159%)**	**-**

### SETS vs ETE

A single study^7^ was unique because it directly compared ETE transfer against SETS transfer through a retrospective cohort in patients with ulnar nerve compression at the elbow. Results are presented separately and were not pooled. An ETE transfer was performed if the patient presented with complete intrinsic muscle atrophy. A SETS transfer was performed if the patient presented with some intrinsic function. Thirty-two nerve transfers were performed including 15 ETE and 17 SETS. The average patient age was 58.3 years, and 78% were male. Type of injury was compression in all cases at the elbow. The average symptom duration was 15.6 months. All 32 patients had pre-operative numbness (Table 8).

**Table 8:** Showing patient demographics, type of injury, level of injury, and pre-operative symptoms. Pre-operative (p).

Study	No of Transfers	Age	M:F	Type of Injury	Location of Injury	Symptom Duration	pWeakness	pNumbness	pPain	pAtrophy	pPositive Froment's Sign
McLeod^7^	32 15 ETE 17 SETS	58.3	25:7	32 compression	32 elbow	15.6 months	15	32	NR	32	15

The average follow-up period was 12 months; however, exact time-to-recovery was not stated. The overall post-operative BMRC score was 2.9 amongst both interventions. When interventions took place before 12 months, the BMRC score was 3.7, whereas the BMRC score was 2.2 amongst interventions performed at or beyond 12 months. This was statistically significant (p < 0.01). The post-operative BMRC score in the SETS group was 3.2 versus 2.6 in the ETE group; however, this was not statistically significant. The subgroup analysis was performed comparing the impact of time-to-transfer on post-operative intrinsic function. When ETE transfer was performed in <12 months, BMRC = 3.4 versus BMRC = 1.9 when performed ≥12months. When SETS was performed in <12 months, BMRC = 4.0 versus BMRC = 2.6 when performed ≥12 months (Table 9). This subgroup analysis was not tested for significance. All patients at final follow-up had a positive Froment's sign, despite the type of transfer performed. A single patient in the cohort developed complex regional pain syndrome (CRPS).

**Table 9:** Showing follow-up period and post-operative outcomes amongst the two cohorts. ETE transfer is performed if patients presented with complete intrinsic muscle atrophy. SETS transfer was performed if patients presented with some intrinsic function. * denotes statistical significance, p < 0.01. Complex regional pain syndrome (CRPS) and no result (NR).

Study	Follow-up (months)	Time to nerve transfer	Intrinsic recovery from symptom onset to surgery	Time to recovery(months)	ETE intrinsic recovery from symptom onset to surgery	SETS intrinsic recovery from symptom onset to surgery	Complications
McLeod^7^	12	15.6 months	Overall BMRC = 2.9 <12 months BMRC = 3.7 ≥12 months BMRC = 2.2* SETS BMRC = 3.2 ETE BMRC = 2.6	NR	<12 months BMRC = 3.4 ≥12months BMRC = 1.9	<12 months BMRC = 4.0 ≥12 months BMRC = 2.6	1 CRPS

### Risk of Bias and Quality Assessment

Using the NIH Quality Assessment Tool for case series, 11 studies were rated ‘fair’ and 1 ‘good’. The single RCT was rated ‘fair’ using the NIH Quality Assessment Tool for controlled intervention studies. Amongst the 3 retrospective cohort studies, scores were 5, 7, and 9 using the Newcastle-Ottawa scale and only single study matched cohorts^13^. None of the authors in any of the articles disclosed any conflicts of interest; however, 3 articles reported an external funding source^18^^,^^25^^,^^26^. There were no clear sources of bias identified.

## Discussion

Ulnar nerve injuries, especially high-level injuries, carry an extremely poor prognosis in terms of functional recovery. The role of a supercharged end-to-side transfer is to preserve the distal motor endplates (‘babysit’) until the native axons can regenerate. Additionally, the donor axons augment the regenerating axons. Traditionally, end-to-end transfers of the AIN to the motor branch of the ulnar nerve were performed for high ulnar nerve injuries^4^^,^^27^. However, the target muscles were only innervated by the donor nerve and not the native ulnar nerve. The SETS transfer allowed end-side coaptation through a perineural window of the damaged recipient nerve allowing axons to sprout and reinnervate distal targets in a shorter time frame as well as double innervation of the motor end plates while the proximal native nerve regenerates. This concept was proven in animal models^28^, ^29^, ^30^. Barbour et al.^5^ were the first to report their results using a SETS AIN to ulnar nerve transfer. Koriem et al. reported that a SETS transfer added only 20-40 minutes of additional operating time compared with an isolated ulnar nerve repair^24^. Sukegawa et al. in their anatomical study found that the mean number of fascicles and axons at the divided end of the pronator quadratus branch of the AIN was 1.2 (1-2) and 506 (372-602), respectively. The mean number of fascicles and axons at the divided end of the deep branch (motor) of the ulnar nerve was 7.8 (6-11) and 1523 (982-2562), respectively^31^. This disparity favours additional reinnervation from more proximal axons and may also account for cases of incomplete recovery. The presence of the distal AIN is normally quite reliable in the absence of any previous trauma. Dy et al. report a case in which the pronator quadratus muscle was absent precluding the use of its nerve as a donor^32^. Such variations should be considered in the decision-making algorithm.

For homogeneity, end-to-end and end-to-side transfers were analysed independently. In the end-to-end group, post-procedure, 100% of patients (37/37) recovered the intrinsic function of BMRC ≥1, and the average recovery time was 3.6 months. About 85% (3 studies) of patients recovered the intrinsic function of BMRC ≥3. In the end-to-side group, 93% (145/156) recovered the intrinsic function of BMRC ≥1, and the average time to recovery was 7 months (4 studies). About 75% of patients recovered the intrinsic function of BMRC ≥3 in their first dorsal interossei. Eleven patients did not recover any intrinsic function. In both groups, there was a high success rate of intrinsic recovery within 12 months with very low morbidity, showing that the AIN to ulnar nerve transfer is a reliable procedure at restoring intrinsic function. Despite improvement in intrinsic function, over an average follow-up period of 19 months, Arami et al. reported no improvement in claw deformity in any of their 11 patients undergoing end-to-end transfers for high ulnar nerve injuries^12^.

In both the SETS and ETE groups, pre-operative mean BMRC was 1.3 and 0, respectively. Post-intervention BMRC was 3.2 and 3.1 in the SETS and ETE groups, respectively, once again showing the success of nerve transfers in helping intrinsic recovery.

The study by McLeod et al.^7^ was unique because it directly compared the results of ETE and SETS in patients with ulnar nerve compression at the elbow. The overall post-operative BMRC score was 2.9 amongst both interventions. When interventions took place in under <12 months, the BMRC score was 3.7, whereas the BMRC score was 2.2 amongst interventions performed above ≥12 months. This result was statistically significant. The post-operative BMRC score in the SETS group was 3.2 versus 2.6 in the ETE group; however, this was not statistically significant. In all cases, BMRC was higher in patients who had interventions performed within 12 months of symptom onset elucidating to the fact that time is a critical factor in motor recovery.

Barbour et al.^5^ proposed SETS for Sunderland grade II and III injuries and ETE transfer for grade IV and V injuries. However, it seems that the SETS transfer has gained more popularity recently because of the advantage of allowing for reinnervation from the proximal repair site. In patients with concomitant or previous AIN injury or peripheral neuropathy, SETS transfer is not beneficial^16^. Electrodiagnostic studies show that absent compound muscle action potentials (CMAP) are a major predictor of poor intrinsic muscle recovery. This signifies the inability of a muscle with severe and prolonged denervation to be reinnervated regardless of axonal regeneration^16^. Power et al.^33^ describe their clinical indications for a SETS transfer for cubital tunnel syndrome, which remains controversial because of its efficacy and timing. The key considerations are the degree of ulnar axonal loss, the quality of recipient intrinsic muscles, and the availability of a normal functioning AIN. Patients with cubital tunnel syndrome who would benefit from a SETS procedure have reduced compound muscle action potential amplitude (indicating axonal loss) and the presence of fibrillation potentials or positive sharp waves on electromyography (indicating that the muscles remain receptive to reinnervation)^33^.

More recently, Felder et al.^34^ described their technique to restore sensation to the vulnerable ulnar border of the hand using allograft or autograft to perform side-side sensory nerve grafting from the median nerve to the ulnar nerve in the palm in conjunction with a SETS AIN transfer. Of the 24 patients who had adequate follow-up to be included, 21 patients (87%) had a return of protective sensation within 1 year. In nerve autograft patients, sensation was found to be referred to the median nerve distribution, and recovery was significantly improved compared with other cohorts. Cross palm sensory nerve grafting may be a useful adjunct to address sensory recovery in severe ulnar nerve neuropathy.

The major limitation of this review was the lack of standardised outcome measures across the studies, with validated scores making comparisons between studies difficult. Some of the outcome measures are highly subjective thus potentially introducing bias. The majority of studies are retrospective case series, with varying indications for nerve transfers including transections and compression neuropathies. Concomitant procedures such as nerve releases were not accounted for which may influence outcomes independently, and surgical techniques also varied across studies such as the use of autologous nerve grafts. Post-operative rehabilitation protocols also differed.

There is a significant lack of high-quality randomised control trials in this exciting field of peripheral nerve surgery. A group in Boston is currently performing a randomised control trial comparing cubital tunnel release with supercharged end-to-side anterior interosseous nerve transfer to a cubital tunnel release alone in patients with severe cubital tunnel syndrome. The primary outcome measure is key pinch strength. Secondary outcomes include two validated patient-reported outcome measures and forearm pronation strength^35^. Results of this study trial are highly anticipated.

## Conclusion

AIN to ulnar nerve transfers carry low morbidity, and there is low-quality evidence (level IV) to suggest superior recovery of intrinsic muscle function compared with conventional primary repair techniques/nerve releases in Sunderland classification II to V ulnar nerve injuries. The supercharged end-to-side transfer (SETS) seems to be more favourable compared with end-to-side transfer because of the potential reinnervation from the proximal ulnar nerve. Outcome measures are highly variable across studies making standardisation difficult. Future research should aim to standardise outcomes using validated patient-reported outcome measures and electrodiagnostic tests. Results of future trials and case series in this exciting field of peripheral nerve surgery are highly anticipated.

## No ethical approval required

No funding to declare.

No conflict of interest to declare.

## Appendix Supplementary materials

Image, application 1
