# Supplementary material for: Anterior Interosseous Nerve to Ulnar Nerve Transfer: A Systematic Review
Source: JPRAS Open. 2022 Mar 11;32:195–210. doi: 10.1016/j.jpra.2022.02.007 (PMC9043848; doi:10.1016/j.jpra.2022.02.007)
Supplement: Supplementary file 1 [file mmc1.docx]

**Appendix 1**

**NIH Quality Assessment Tool for Case Series Studies**^9^

| Criteria | **Battiston**^14^ | **Haase 2002**^21^ | **Novak 2002**^4^ | **Flores 2011**^19^ | **Davidge 2015**^16^ |
| --- | --- | --- | --- | --- | --- |
| 1. Was the study question or objective clearly stated? | Y | Y | Y | Y | Y |
| 2. Was the study population clearly and fully described, including a case definition? | Y | Y | Y | Y | Y |
| 3. Were the cases consecutive? | N | N | N | N | N |
| 4. Were the subjects comparable? | Y | Y | Y | Y | Y |
| 5. Was the intervention clearly described? | Y | Y | Y | Y | Y |
| 6. Were the outcome measures clearly defined, valid, reliable, and implemented consistently across all study participants? | Y | N | Y | Y | N |
| 7. Was the length of follow-up adequate? | Y | Y | Y | Y | N |
| 8. Were the statistical methods well-described? | N | N | N | N | Y |
| 9. Were the results well-described? | Y | Y | Y | Y | Y |
| Quality rating (good, fair, and poor) | Fair | Fair | Fair | Fair | Fair |

| Criteria | **Doherty 2018**^18^ | **Jarvie 2018**^23^ | **Arami 2020**^12^ | **Dengler 2020**^17^ | **Head 2020**^22^ |
| --- | --- | --- | --- | --- | --- |
| 1. Was the study question or objective clearly stated? | Y | Y | Y | Y | Y |
| 2. Was the study population clearly and fully described, including a case definition? | Y | N | Y | Y | Y |
| 3. Were the cases consecutive? | Y | N | N | N | N |
| 4. Were the subjects comparable? | Y | Y | Y | Y | Y |
| 5. Was the intervention clearly described? | Y | Y | Y | Y | Y |
| 6. Were the outcome measures clearly defined, valid, reliable, and implemented consistently across all study participants? | Y | Y | Y | Y | Y |
| 7. Was the length of follow-up adequate? | Y | Y | Y | Y | Y |
| 8. Were the statistical methods well-described? | Y | N | Y | Y | Y |
| 9. Were the results well-described? | Y | Y | Y | Y | Y |
| Quality rating (good, fair, and poor) | Good | Fair | Fair | Fair | Fair |

| Criteria | **McLeod 2020**^7^ | **Nyman 2021**^25^ |
| --- | --- | --- |
| 1. Was the study question or objective clearly stated? | Y | Y |
| 2. Was the study population clearly and fully described, including a case definition? | Y | Y |
| 3. Were the cases consecutive? | N | N |
| 4. Were the subjects comparable? | Y | Y |
| 5. Was the intervention clearly described? | Y | Y |
| 6. Were the outcome measures clearly defined, valid, reliable, and implemented consistently across all study participants? | Y | Y |
| 7. Was the length of follow-up adequate? | N | Y |
| 8. Were the statistical methods well-described? | Y | N |
| 9. Were the results well-described? | Y | Y |
| Quality rating (good, fair, and poor) | Fair | Fair |

**Newcastle-Ottawa scale**^8^

|  | **Selection** | | | | **Comparability** | **Outcome** | | |  |
| --- | --- | --- | --- | --- | --- | --- | --- | --- | --- |
|  | Representativeness  of exposed  cohort | Selection of  non-exposed  cohort | Ascertainment of  exposure | Outcome of  interest not  present at start  of study | Comparability  of cohorts | Assessment of  outcome | Follow-up  long enough  for outcomes  to occur | Adequacy of  follow-up of  cohorts | **Score** |
| Flores 2015^20^ | **🟑** | **🟑** | **🟑**  Electronic record | **🟑** | No matching | **🟑**  Electronic record | **🟑**  24.3 months | **🟑**  12.5% lost to follow-up | 7 |
| Baltzer 2016^13^ | **🟑** | **🟑** | **🟑**  Electronic record | **🟑** | **🟑🟑**  Matched | **🟑**  Electronic record | **🟑**  13.5 months | **🟑** | 9 |
| Chen 2021^15^ | **🟑** | **🟑** | No description | **🟑** | No matching | No description | **🟑**  12 months | **🟑** | 5 |

**NIH Quality Assessment Tool for Controlled Intervention Studies**^9^

|  | **Koriem**^24^ |
| --- | --- |
| 1. Was the study described as randomized, a randomized trial, a randomized clinical trial, or an RCT? | Y |
| 2. Was the method of randomization adequate (i.e., use of randomly generated assignment)? | Y |
| 3. Was the treatment allocation concealed (so that assignments could not be predicted)? | N |
| 4. Were study participants and providers blinded to treatment group assignment? | N |
| 5. Were the people assessing the outcomes blinded to the participants' group assignments? | N |
| 6. Were the groups similar at baseline on important characteristics that could affect outcomes (e.g., demographics, risk factors, and co-morbid conditions)? | Y |
| 7. Was the overall drop-out rate from the study at endpoint 20% or lower of the number allocated to treatment? | Y |
| 8. Was the differential drop-out rate (between treatment groups) at endpoint 15 percentage points or lower? | N |
| 9. Was there high adherence to the intervention protocols for each treatment group? | Y |
| 10. Were other interventions avoided or similar in the groups (e.g., similar background treatments)? | Y |
| 11. Were outcomes assessed using valid and reliable measures, implemented consistently across all study participants? | Y |
| 12. Did the authors report that the sample size was sufficiently large to be able to detect a difference in the main outcome between groups with at least 80% power? | N |
| 13. Were outcomes reported or subgroups analyzed prespecified (i.e., identified before analyses were conducted)? | N |
| 14. Were all randomized participants analyzed in the group to which they were originally assigned, i.e., did they use an intention-to-treat analysis? | Y |
| Quality rating (good, fair, and poor) | Fair |
